# Supplementary material for: Are carbon emissions trading and green financial instruments synergistic? -Comprehensive quantitative research based on content analysis
Source: PLoS One. 2024 Mar 7;19(3):e0298601. doi: 10.1371/journal.pone.0298601 (PMC10919601; doi:10.1371/journal.pone.0298601)
Supplement: S1 Data — (ZIP) [file pone.0298601.s001.zip › Data/地级市Z_F工作报告.zip!/║╙─╧/╫ñ┬φ╡Ω╩╨/└┤╫╘╣1⁄2╓┌║┼í╛┬φ┐╦╩2╛▌═°í┐_2006.docx]

各位代表:

现在，我代表市人民政府，向大会作工作报告，请予审议，并请各位政协委员提出意见。

折叠编辑本段2005年工作回顾

折叠项目建设力度加大，工业经济快速发展

全年计划安排101个重点项目，实际实施118个，完成投资102.4亿元，为年度计划的106%。其中40个计划竣工项目完成投资27.1亿元，为年度计划的91.9%;27个续建项目完成投资65.3亿元，为年度计划的110.8%;21个计划开工项目实际开工37个，开工率176%。在建项目数量和投资增幅均为历史最高。坚持把工业项目作为重中之重，73个重点工业项目完成投资55.9亿元，为年度计划的106%。蓝天集团年产40万吨甲醇、豫龙水泥日产5000吨熟料生产线、骏马化工三聚氰胺、鲁洲生物科技一期、益康面粉二期等项目已竣工投产;蓝天集团燃气电厂、华润古城燃煤电厂、天方药业合成分厂、吴桂桥煤矿、华骏工业园二期、惠丰生物科技等一批重点项目，今年将全部投产。同时，注重引导企业深化改革，加强管理，促进了工业经济的快速发展。全市限上工业完成增加值76.1亿元，增长24%，增速为历史最高;完成销售收入215亿元，增长38.9%;实现利润11.4亿元，增长77.6%;实现利税21.9亿元，增长47.6%;综合效益指数157.9%，比上年提高25.1个百分点。

折叠"三农"工作得到加强

加快了农业综合开发和7个县优质小麦基地建设，全市优质专用小麦达到688万亩，占麦播面积的74.5%，粮食总产532万吨，虽因灾减产，但仍为历史上第二个丰收年。畜牧业增加值达到73.3亿元，增长7.6%，肉、蛋、奶产量进一步提高。完成成片造林20.25万亩，新建完善农田林网359万亩，实现了全市平原绿化高级达标。实施了小流域治理、灌区配套、平原打井等重点水利工程，新增有效灌溉面积32.9万亩、旱保田30万亩，改造中低产田21万亩。因地制宜发展特色经济，扩大了优质专用小麦和经济作物生产规模，培育壮大了一批农畜产品加工龙头企业，加快了农业产业化进程。大力发展劳务经济，全年输出劳务181万人次，实现劳务收入60亿元。全市县域生产总值达到411亿元，增长11.5%;县域工业增加值132亿元，增长16.2%;县级财政一般预算收入8.5亿元，增长29.5%。

折叠城镇建设步伐加快，城镇化水平提高

全年中心城区完成建设总投资9.26亿元，增长38.2%，会展中心、天中广场、解放大道西段、风光路南段、前进路北段、雪松大道东段等项目已建成使用;开元河公园一期、交通路西段改造等项目进展顺利，行政新区建设取得了实质性进展。深入开展"三城联创"活动，加强城市环境综合整治，市区经营秩序、市容市貌、环境卫生发生了显著变化，中心城区绿化覆盖率达到24.7%。县城和小城镇建设步伐加快，全市城镇化率达到18.67%。

折叠改革开放稳步推进，招商引资取得新成效

国有企业改制工作基本完成。粮食流通体制改革任务全面完成，新成立国有独资企业12家、股份制企业87家。农信社改革取得阶段性成效，10个县区全部一次性拿到央行专项票据。乡镇机构改革扎实推进，顺利完成了乡镇撤并和人员分流任务。行政审批及交通、城建、商贸等领域的改革，也都迈出了新步伐。加大招商引资力度，成功举办了第八次全国东西合作经贸洽谈会，全年共落实签约项目416个，投资总额65.8亿元，其中引进资金63亿元，促成了中国昊华、山东鲁洲、中集集团、香港华润、日本住友、英国爱农等一批国内外知名企业落户我市。全年实际利用外资3134万美元，完成出口创汇5060万美元，分别增长30.6%和11.2%。

折叠社会事业全面进步，经济社会协调发展

阿深、上武、新蔡-泌阳3条高速累计完成投资24.7亿元，干线公路完成投资6.9亿元，建设干线公路262.8公里。组织实施科技项目214项，转化科技成果132项，科技对经济增长的贡献率达到46%。教育"两基"成果得到巩固，农村办学条件进一步改善;高中在校生达到13.8万人，又有4所学校被认定为省级示范性高中;引进资金6.8亿元，新建各类民办学校10所;黄淮学院对外合作办学取得突破。卫生事业全面进步，实施疾病预防和医疗救治体系项目21个，建设改造乡镇卫生院66所，艾滋病防治救助工作取得新的成效。计划生育工作得到加强，人口自然增长率5.4‰，低于全省平均水平。实施文化精品工程，全年创作大剧本4部。广播电视、新闻出版、群众体育和竞技体育健康发展。环保执法力度加大，环境质量继续改善。深入开展拥军优属、拥政爱民和军民共建活动，军政军民关系进一步密切。旅游、统计、人事、审计、监察、气象、人防、民族宗教、史志、档案、侨务等工作都取得了新成绩。

折叠坚持以人为本，认真解决人民群众的切身利益

去年，向全市人民承诺的15件实事得到较好落实。全年新增就业人员46639人，实现下岗失业人员再就业18777人，其中"4050"人员5235人，占省定目标的194%。企业职工基本养老保险实现市级统筹，国有企业下岗职工基本生活费、失业人员保险金、企业离退休人员养老金实现了按时足额发放;为10.3万城市低保对象发放低保金6463万元，做到了应保尽保。全面落实了免征农业税、对种粮农民直补、良种补贴和大型农机具购置补贴政策，"一免三补"使全市农民减负增收5.62亿元，人均81元;全年解决温饱人口3万人，巩固温饱人口8万人。对农村困难家庭学生发放"两免一补"资金1.41亿元，资助学生130.4万人;筹措资金2.05亿元，完成了480个行政村、1280公里的"村村通"建设任务;筹措资金6200万元，解决了317个自然村27.8万群众的安全饮水问题;筹措资金6796万元，实施农村中小学D级危房改造项目307个。切实做好灾区倒房重建工作，政府帮建新房8488间。加大清理建设领域拖欠工程款和农民工工资力度，维护了进城务工人员的合法权益。

折叠精神文明和民主法制建设得到加强

社会大局稳定认真开展保持共产党员先进性教育活动，切实加强思想道德建设，积极推进文明城市、文明单位、文明村镇等精神文明创建活动，城乡面貌和干部群众的精神风貌发生了显著变化。坚持依法治市，深入开展普法教育，完成了"四五"普法任务。大力推进村务、政务公开，顺利完成了第五届村委换届选举工作。自觉接受人大法律监督和政协民主监督，认真办理人大代表建议和政协提案。深入开展"平安建设"活动，加强社会治安防控体系建设，严厉打击各种违法犯罪，刑事案件立案数同比下降31.1%，命案侦破率位居全省前列。全面落实安全生产责任制，全年安全事故率和死亡率分别下降20.5%和22.9%。认真贯彻《信访条例》，积极排查化解矛盾，依法妥善处置群体性事件，赴京、赴省上访批数明显下降，保持了社会大局稳定。

各位代表，过去的一年是强力推进"三项建设"、实现驻马店平安崛起迈出坚实步伐的一年，是全市人民群众得到实惠最多的一年。一年来的成绩，是省委、省政府和市委正确领导的结果，是市人大、政协积极支持的结果，是全市广大干部群众共同奋斗的结果。在此，我代表市人民政府向工作在全市各条战线的广大工人、农民、知识分子、干部、驻驻部队指战员、武警官兵、政法干警和社会各界人士致以崇高的敬意!向所有关心支持政府工作的各位人大代表、政协委员和民主党派、工商联、无党派人士表示衷心的感谢!

在肯定成绩的同时，我们也清醒地看到，当前我市经济社会发展中还存在不少矛盾和问题:经济总量小，综合实力不强，区域竞争压力较大;经济结构不合理，二产比重偏低，工业骨干企业少，对经济发展的支撑作用不强;农业基础薄弱，农业产业化水平不高，粮食增产和农民增收难度加大;城镇化水平低，就业压力大，城乡发展不平衡性比较突出;财政收入结构不合理，财政困难状况没有得到根本改变;经济发展环境有待进一步改善;一些群众的生产生活还十分困难，看病贵和上学难的问题比较突出，群众反映强烈。对此，我们一定进一步增强使命感和责任感，以更加务实的作风，努力把政府各项工作做得更好，绝不辜负全市人民的重托和厚望。

折叠编辑本段2006年政府工作的主要目标和任务

2006年是"十一五"规划的开局之年。今年我市经济社会发展的主要预期目标是:全市生产总值增长12%，限上工业增加值增长25%，全社会固定资产投资增长30%，财政一般预算收入增长12%，社会消费品零售总额增长13%，城镇居民人均可支配收入增长8%，农民人均纯收入增长6%，城镇新增就业4.2万人，城镇登记失业率控制在4.5%以内，人口自然增长率控制在6‰以内。实现上述目标，要重点抓好以下八个方面的工作:

折叠大力发展工业经济

突出抓好工业项目建设。今年初步安排重点工业项目114个，总投资213.6亿元，年度计划投资57.04亿元。蓝天集团燃气电厂、华润古城燃煤电厂、天方药业阿奇霉素原料药、吴桂桥煤矿一期、华骏工业园二期等73个计划竣工项目，确保按期竣工投产。骏马化工年产2万吨DMF及2万吨甲胺、河南顺达化工年产20万吨醋酸、华骏铸造二期等16个续建项目，确保完成年度投资计划。豫龙水泥、白云纸业二期等19个计划新开工项目，确保按期开工建设。同时，抓好热电厂2×30万千瓦热电联产、蓝天集团20万吨生物柴油和骏马化工合成氨原料路线改造等6个项目的前期工作，并围绕我市的产业优势，积极论证储备一批项目，争取更多项目纳入国家和省计划。加大对重点企业的扶持力度。从今年起，筛选一批具有发展潜力、带动性强的企业，作为重点扶持企业，整合出台相关优惠政策，完善有关激励约束机制，全方位营造良好的发展环境，促进企业做大做强，争取天方药业、蓝天集团年销售收入突破50亿元，骏马化工达到30亿元，华骏车辆、南方钢铁达到10亿元以上。继续推进工业结构调整。加大自主创新力度，实施一批结构调整和技术改造项目，促进工业结构升级。围绕资源优势，大力发展农畜产品和矿产资源精深加工业。支持优势企业、工业园区和产业集群的发展，努力提高产业聚集度。加强对工业运行的协调指导。搞好工业运行监测，做好电力、煤炭、运力的供求衔接，定期研究解决工业运行有关问题。同时引导企业加强内部管理，强化市场营销，抓好扭亏增盈，促进工业经济快速发展。

折叠扎实推进社会主义新农村建设

继续加强"三农"工作，全面落实各项支农惠农政策，力争新农村建设取得良好开端。积极推进现代农业建设。继续抓好7个优质小麦基地县、5个农业综合开发重点县和2个世行贷款县项目建设，进一步提高粮食综合生产能力。大力培育和壮大龙头企业，重点抓好天乳奶业、丰盛粮油等一批农畜产品加工项目建设，积极推进优势农畜产品的区域化布局、规模化生产，加快农业产业化进程。高度重视禽流感防控工作，加强动物疫病防控体系建设，全面完成县级防疫站建设任务。加强林业生态建设，完成成片造林15万亩，新建完善通道绿化500公里，巩固全市平原绿化高级达标成果。积极发展劳务经济，强化组织引导，加强技能培训，力争培训农村劳动力7.8万人，新增转移就业15万人。加大整村推进扶贫开发力度，确保11万农村贫困人口脱贫和解决温饱。加强农村基础设施建设。加快小洪河、大洪河治理和老王坡滞洪区安全工程建设，开工建设薄山水库、板桥水库灌区节水改造配套工程，继续抓好农村节水灌溉和山区小流域治理，力争新增有效灌溉面积20万亩、旱保田20万亩，治理水土流失50平方公里。积极争取省"村村通"专项资金，确保完成600个行政村、1600公里的"村村通"工程。扶持农村沼气工程建设，完成省定目标任务。抓好农村安全饮水工程，确保解决12万农村人口的安全饮水问题。多渠道筹措资金，加大农村教育、文化、卫生等社会事业的投入，改善农村中小学办学条件，加快农村乡镇卫生院改造，实施广播电视"村村通"工程，不断满足广大群众对卫生保健和精神文化的需求。全面深化农村改革。推进乡镇机构职能转变和服务水平提高，深化农村义务教育和县乡财政体制改革，继续实行对种粮农民直补、良种补贴和大型农机具购置补贴，巩固农村税费改革成果，增强农村发展活力。加强乡村规划和文明建设。立足发展基础和乡村特色，因地制宜制定乡村规划，量力而行实施一批乡村建设示范工程。注重村容村貌整治，积极培育造就新型农民，倡导健康文明的新风尚，不断提高乡村的文明程度。扩大农村基层民主。搞好村民自治，健全村务公开制度，开展普法教育，规范"一事一议"，促进农村社会和谐。

折叠进一步加快城镇化进程

突出抓好中心城区建设。加快推进行政区北迁工程，抓好东三环、通达路、交通路西段、练江路东段、骏马路北段、驿城大道等市区道路建设，继续改善城区通行条件。完善优惠政策，实施连片开发，进一步加大旧城拆迁和改造力度。加快通达住宅小区、练江堡住宅小区等房地产项目和市垃圾处理厂建设。深化"三城联创"活动，努力实现创建目标。加快县城和小城镇发展。引导县城和重点镇，立足现有基础，完善城镇功能，培育支柱产业，扩大规模，提升辐射带动能力。深化户籍制度和投融资体制改革。积极消除有关体制和政策障碍，促进人口向城镇聚集。积极引导社会资金参与城镇供水、供热、污水治理、垃圾处理等公用设施建设，通过公开拍卖城市黄金地段开发权、广告发布权和道路桥梁冠名权，搞活城市经营。大力发展县域经济。进一步落实各项政策措施，完善县城基础设施和公共服务，大力发展特色经济、园区经济、劳务经济，努力构建以工业化为主攻方向、以民营经济为发展支撑的县域经济格局，推进县域经济发展再上新台阶。加强交通建设。加快上武高速泌阳段、棠西路、平桐路、吴磺路等项目建设进度，确保年内建成通车;抓好新阳高速、驿阳高速、阿深高速驻马店段等续建项目建设;做好安信快速通道开工建设工作，支撑城镇加快发展。

折叠加大改革开放力度

继续推进国有企业改革。加快银马纺织、柴油机厂、龙山水泥厂等企业改制步伐，全面完成改制任务;加大已改制企业的重组力度，积极引进战略投资伙伴，努力实现股权多元化。加快供销、商贸、交通、城建、水利等局办企业改革，完成年度目标任务。继续完善国有资产有效监管体制，确保国有资产保值增值。进一步深化财政改革。继续落实政府采购制度，扩大采购范围和规模，继续探索市县联合等采购新模式，提高财政资金使用效益。完善国库集中支付制度，加强财政监督，确保财政资金运行安全高效。积极发展资本和其他要素市场。支持具备条件的企业上市和上市公司再融资，扩大债券融资规模。改善金融生态环境，维护金融债权，规范企业贷款中介行为，加快建设社会信用体系。加强人才、技术、产权交易市场建设。全面推进其他领域的改革。巩固粮食流通、农村信用社改革成果，深化供销社体制改革，增强服务功能。加大小型水利设施产权制度改革力度。加快交通、城建、市政公用事业改革步伐。积极推进卫生、科技、教育等领域的改革。

坚持以东西合作为载体，进一步扩大对外开放。继续加大招商引资力度。围绕资源和产业优势，有重点地开展招商活动，提高招商引资实效。创新招商方式，多渠道引进国内外资金。加快工业集中区建设，完善基础设施，实现生产要素的快速聚集。建立完善代理制和"两个零接触"制度，大力营造浓厚的亲商、安商、富商氛围。继续办好东西合作经贸洽谈会，进一步提高办会效果。巩固招商引资成果。重点抓好已签约项目的履约工作，明确专人负责，搞好跟踪问效，促使签约项目落实、合同引资到位。大力发展外向型经济。继续扩大利用外资规模，提高利用外资水平，力争实际利用外资增长25%以上。重点支持天方药业、华中正大、骏马化工、华骏车辆、康茂、荣光等企业扩大工业品出口，继续抓好优质小麦、芝麻、生猪等优势农产品出口。

折叠推进第三产业快速发展

大力发展服务业。坚持运用现代经营方式和信息技术手段，对传统服务业进行功能结构、网点布局调整，完善配套设施，推进体制创新，提高经济效益。坚持市场化、产业化方向，积极发展房地产、金融、保险、信息和法律服务等现代服务业。加大资金投入，改善社区服务条件，完善社区就业和文化、卫生服务网络，促进社区服务业发展。积极发展文化产业。加快国有文化企业公司制改造和经营性文化事业单位改制，培育多元化经营主体。加强政策扶持，实施资源整合，组建天中广电传媒有限公司。加大文化基础设施建设力度，筹建市广播电视中心、报业大厦、图书馆、群艺馆、体育馆、工人文化宫。加快旅游业发展。推进旅游景区企业化经营、市场化运作，提高旅游综合效益。突出抓好嵖岈山、薄山湖、铜山湖生态旅游和竹沟红色旅游、天中山生态文化游精品景区建设，加强宣传推介，优化旅游环境，提升旅游业发展水平。

折叠统筹社会事业协调发展

大力发展科技事业。注重先进适用技术的引进、推广和应用，加强与科研院所和大专院校的合作，鼓励骏马化工、中泰药业等企业建立研发中心，支持天方药业、蓝天集团、华骏车辆等企业申报承担国家、省级研发任务，不断增强自主创新能力。优先发展教育事业。搞好农村中小学布局调整，做好农村中小学危房改造和"两免一补"工作，加大对农村义务教育投入，确保农村中小学公用经费落到实处;大力发展高中教育、职业教育和民办教育，积极发展高等教育，争取新增省级示范性高中2-3所，"驻马店职业技术学院"筹建工作取得实质性进展，黄淮学院招生规模继续扩大。加快发展卫生事业。深化医疗卫生体制改革，整顿和规范医疗服务秩序，加强医院管理，扩大农村新型合作医疗试点，认真解决群众看病难、看病贵问题。切实做好艾滋病防治救助工作，确保防治救助取得更大成效。加强环境保护。抓好洪、汝河和黑河水污染防治。确保西平、平舆等7个县城污水处理厂建成投运，遂平、上蔡2个县城污水处理厂按期开工。积极推行清洁生产，综合治理城市环境，提高城市绿化率和垃圾无害化处理率。强化对土地、森林等自然资源的生态保护，重视资源节约工作，积极发展循环经济，努力构建资源节约型和环境友好型社会。支持国防建设。加强国防教育，做好国防动员、人民防空和民兵预备役工作，落实军人优抚和退伍安置政策，巩固"双拥"成果。抓好计划生育等方面工作。全面落实一把手负责制和一票否决制，继续稳定低生育水平。深化统计制度改革，加强基层统计建设，搞好第二次全国农业普查。依法保护妇女、儿童、未成年人和残疾人的合法权益。做好审计、监察、人事、外事侨务、史志、气象、红十字会等工作。

折叠努力构建和谐社会

全面落实好今年向全市人民承诺的15件实事，解决好关系广大群众的切实利益问题，促进社会和谐稳定。做好就业再就业工作。大力发展劳动密集型企业、社区服务业和民营经济，努力增加就业岗位;全面落实小额贷款、税费减免等再就业扶持政策，鼓励下岗失业人员自主创业和自谋职业，力争新增就业人员4.2万人，下岗失业人员再就业1.1万人，其中"4050"人员4000人以上。加强社会保障体系建设。扩大城镇社会保险覆盖面，加大征缴力度，完善城镇职工基本养老保险制度，确保养老金按时足额发放。完善城市居民最低生活保障制度，实现动态管理下的应保尽保。加强城镇基本医疗、工伤、生育保险制度建设。落实好农村特困户救助和"五保户"生活保障制度，提高农村特困人口的生活补助标准。解决好群众关心的热点、难点问题。加强对食品药品、饮食卫生等行业的监管，保护群众生命安全。规范土地征用、城市拆迁、企业改制工作，切实保护城乡居民的合法权益。继续开展"平安建设"活动，加强社会治安综合治理，完善社会治安防控体系，严厉打击各类违法犯罪，保持社会治安大局稳定。加强安全生产监管，认真落实安全生产责任制，防止重特大事故发生。高度重视群众来信来访，加强基层基础工作，认真解决人民群众反映强烈的问题。抓好精神文明建设。加强理想信念教育和公民道德建设，不断提高全民思想道德素质。深入开展文明城市和文明单位、文明村镇、文明社区等精神文明创建工作，不断提高城乡文明程度。积极开展科技、文化、卫生"三下乡"活动，破除封建迷信，深化殡葬改革，倡导文明健康的生活方式，营造文明和谐的社会环境。推进民主法制建设。认真执行人大及其常委会的决议、决定，支持人大代表依法行使职权。加强与政协的政治协商，自觉接受政协的民主监督。认真办理人大代表建议和政协提案。广泛听取各民主党派、工商联和无党派人士的意见。认真贯彻党的民族宗教政策，继续做好民族宗教工作。大力推进政务、厂务、村务公开，进一步完善基层民主管理制度。加强法制宣传教育，推进依法治市进程。

折叠加强政府自身建设

面对新的形势和任务，我们必须加强政府自身改革和建设，努力做到对人民负责、受人民监督、让人民满意。加快职能转变。积极推进行政管理体制改革，加快政企、政事分开，集中精力抓市场主体的培育，抓发展环境的改善。继续减少和规范行政审批事项。进一步改进社会管理，协调好各种利益关系，促进社会公平和正义。完善政府责任目标考评制度，增加衡量经济社会发展的硬性指标，继续实行主要发展指标定期通报制度。提高服务水平。努力改进服务方式，提高服务质量，更好地为基层、企业和社会服务。积极推进电子政务建设，健全行政服务大厅功能，精简办事程序，切实履行服务承诺。充分发挥优化环境110的作用，严厉打击强装强卸、强买强卖、欺行霸市等干扰施工秩序和"四乱"行为，痛下决心优化发展环境。坚持依法行政。认真贯彻国务院依法行政实施纲要和行政许可法。健全对重大事项决策的专家论证制度，完善涉及群众切身利益相关事项的决策公示和听证制度。全面推行领导干部责任审计制度，切实加强行政监察。深入开展廉政建设和反腐斗争，重点治理工程建设、土地出让、产权产易、医药购销和政府采购等领域的商业贿赂问题。加强政风建设。多做打基础、管长远的工作，力戒官僚主义、形式主义。带头厉行节约，反对奢侈浪费，建设节约型机关。继续精简会议、文件，严格控制各种检查评比活动。坚持求真务实，大兴调查研究之风，掌握实情，解决实际问题。加强目标管理和督查，强化工作责任，确保各项工作落到实处。

折叠编辑本段关于"十一五"规划《纲要(草案)》的说明

《驻马店市国民经济和社会发展第十一个五年规划纲要(草案)》(以下简称《纲要(草案)》)，已提请大会审议。下面，我就几个有关问题作简要说明。

折叠编制过程和主要特点

市委、市政府对制定"十一五"规划工作高度重视。2004年9月，市政府下发了《"十一五"规划编制方案》，启动了规划编制的相关工作。组织有关部门和专家，进行专题研究，提出了规划的基本思路。市委一届九次全会以后，根据市委《关于制定国民经济和社会发展第十一个五年规划的建议》精神，深入研究和编制了《纲要(草案)》。随后，在广泛征求社会各方面意见和建议的基础上，聘请有关专家进行了论证，作了多次的修改和完善，提交这次代表大会审议。

《纲要(草案)》依据市委一届九次全会《建议》精神，反映了我市改革发展的新要求，具有宏观性、战略性和政策性。在规划内容上，贯穿了科学发展观的指导思想，突出了发展主题，明确了发展的战略重点和基本任务;在规划指标上，充分体现了加快发展、缩小差距的要求，所定指标既积极稳妥，又符合实际、留有余地;在规划形式上，采取章节结构、正文加专栏的形式，在专栏中列举了有关发展指标和重点项目，使规划重点内容比较突出，便于理解和把握。

折叠国民经济和社会发展主要情况

"十五"是我市改革发展的重要时期。面对日趋激烈的区域竞争压力，我们始终把发展作为第一要务，集中精力解决经济社会发展中的突出矛盾和问题，全面推进工业化、城镇化和农业现代化进程，经济和社会发展取得了显著成就。五年来，我市综合经济实力进一步增强。市内生产总值增加216亿元，年均增长9.2%;限上工业增加值比"九五"末翻了一番多;累计完成财政一般预算收入60.5亿元，为"九五"时期的1.8倍;"二、一、三"产业格局得到巩固;城镇化率比"九五"末提高6.57个百分点。五年来，我市基础设施建设成效显著。完成全社会固定资产投资500多亿元，年均增长21.7%，一大批工业、农林水利、交通、城建、社会事业等项目建成投用。全市新增有效灌溉面积113万亩、公路通车里程880公里、年单向输送天然气16亿立方米、发电装机容量9万千瓦。五年来，我市改革开放取得新的成效。国有企业、行政管理、农村税费、粮食流通等体制改革顺利推进，开放型经济较快发展，东西合作成效明显，出口创汇年均增长29%，五年实际利用外资9631万美元。五年来，我市城乡居民生活明显改善。城镇居民人均可支配收入和农民人均纯收入年均增长9.4%和5.47%。累计安排就业再就业9万余人;实现稳定脱贫84万人。五年来，我市社会事业全面进步。科技对经济增长的贡献率提高6个百分点;完成中小学危房改造756所，完成高中扩建项目26个，黄淮学院申办成功;疾病预防和医疗救治体系建设得到加强，改造建设乡镇卫生院127个、疾病控制中心10个;人口自然增长率低于全省平均水平。总之，经过五年的努力，我市经济社会发展迈上了新的台阶。站在新的起点上，我们更有能力实现又快又好的发展。

折叠发展的指导原则和主要目标

"十一五"时期是我市全面建设小康社会，实现驻马店平安崛起的关键时期。《纲要(草案)》体现了市委《建议》提出的重要原则。这就是:必须坚持把发展作为第一要务，必须坚持加快转变经济增长方式，必须坚持项目带动战略不动摇，必须坚持高度重视"三农"问题，必须坚持以人为本的发展理念，必须坚持以改革开放和自主创新为动力。

根据市委《建议》确定的指导思想和原则，《纲要(草案)》提出了"十一五"时期我市经济社会发展的主要奋斗目标:全市生产总值年均增长12%以上，人均生产总值提前3年实现比2000年翻一番，城镇化水平达到30%以上，全社会固定资产投资年均增长30%以上，城镇居民人均收入、农民人均纯收入年均增长8%和6%以上，人口自然增长率控制在6‰以内。这里，我仅就经济增长速度问题作简要说明。

《纲要(草案)》提出今后五年我市生产总值年均增长12%，比全省预期目标高2个百分点，这是综合考虑各方面因素，根据需要和可能提出的。一方面根据我市现有发展水平，按照全面建设小康社会目标，必须要有一个较高的发展速度，只有这样才能逐步缩小差距;另一方面，近年来我们通过实施项目带动战略，新上了一批规模较大的工业项目，这些项目绝大部分将在"十一五"时期发挥效益。同时，"十一五"期间我市将继续加大项目建设力度，规划建设一批重大工业项目，随着这些项目的相继建成投运，必将拉动我市经济的快速发展。因此，这个速度是积极的，也是符合实际的，通过努力是完全可以实现的。

折叠主要任务

《纲要(草案)》全面提出了"十一五"时期全市经济社会发展的主要任务。这里，我着重说明以下六个方面:

一是加快工业化进程，促进产业结构优化升级。工业化是"三化"的核心，是实现以工补农、以城带乡的关键所在，我们必须把工业摆到更加突出的位置。《纲要(草案)》提出，按照走新型工业化道路的要求，推动产业结构优化升级。强调通过建设一批大项目，扩充工业总量和规模;以信息化带动工业化，改造提升传统产业;有选择地发展高新技术产业，大力发展劳动密集型产业;加快资源整合，着力培育壮大医药、能源和煤化工、轻纺、食品、建材五大支柱产业和一批大型企业集团。这是针对我市工业总量小、骨干企业少、结构层次低、竞争能力不强的现状，提出的一系列重大措施。我们一定要牢固树立工业兴市的理念，大干五年，奋起直追，努力实现我市工业经济的跨越式发展。

二是推进农业现代化，建设社会主义新农村。我市作为农业大市，农村人口多、农业基础较差，农民收入水平低，解决"三农"问题是摆在我们面前的一项重大历史任务。《纲要(草案)》提出，按照生产发展、生活宽裕、乡风文明、村容整洁、管理民主的要求，扎实稳步推进社会主义新农村建设。强调要加大结构调整力度，加快农业产业化步伐，推进农业现代化建设;要加强对农民工的培训，提高劳务输出的组织化程度，大力发展劳务经济;全面推进农村综合改革，认真落实各项支农惠农政策;加大对农业和农村的投入，加快农村基础设施和公共事业发展。我们必须抓住新农村建设的机遇，认真落实相关政策措施，努力开创农村工作新局面。

三是强力推进城镇化，推动城乡协调发展。目前我市城镇化水平比全省低12个百分点，比全国低20多个百分点，加快城镇化发展的任务十分艰巨。针对这一现状，《纲要(草案)》对推进城镇化进程作了具体部署。"十一五"期间我们必须坚持优化城镇布局，强化驻马店中心城市的地位，壮大城镇支柱产业，提高城镇经营管理水平，不断提高吸纳聚集能力，力争城镇化率达到30%以上。发展县域经济是城镇化的重要支撑，也是统筹城乡发展的结合点。《纲要(草案)》还强调要大力发展县域经济。

四是加快基础产业发展，改善经济社会发展的硬环境。近年来，我市加大了基础设施建设力度，为今后发展奠定了基础，但仍然存在不少薄弱环节。"十一五"期间，我们一定要按照《纲要(草案)》的要求，优先发展交通运输、能源、水利等产业，加快影响全局的基础设施建设，从根本上缓解基础设施和基础产业的"瓶颈"制约，切实增强对经济社会发展的支撑和拉动作用。

五是深化改革和扩大开放。二十多年来，我市经济社会发展取得的巨大成就，主要得益于改革开放。要完成新阶段的发展任务，突破影响生产力发展的体制机制障碍，必须通过深化改革和扩大开放来解决。《纲要(草案)》提出，要深化财税金融体制、投资体制、行政管理、事业单位、流通体制和国有企业等重点领域的改革，为加快发展提供强大动力和体制保障。要抓住国内外产业转移的机遇，大力实施东西合作，强力推进招商引资，以开放促改革、促发展。

六是有效推进和谐社会建设。这既是经济社会发展的重要目标，也是经济社会协调发展的重要保障。《纲要(草案)》坚持以人为本，注重人的全面发展，就构建和谐社会有关内容作了较为全面的安排和阐述。"十一五"期间，我们要扩大就业，完善社会保障体系，保护资源和环境，控制人口增长，提高公共安全保障水平，切实解决好涉及人民群众切身利益的问题，继续加强社会主义民主法制建设和精神文明建设。经过五年的努力，使我们的社会更加和谐，让人民群众生活的更加美好!

各位代表,未来的五年，是我们全面建设小康社会、实现驻马店平安崛起的关键时期。新的蓝图已经绘就，让我们以邓小平理论和"三个代表"重要思想为指导，全面落实科学发展观，团结奋进，扎实工作，为顺利实现"十一五"规划目标而努力奋斗!
